# Complete genome sequence of the thermophilic *Thermus* sp. CCB_US3_UF1 from a hot spring in Malaysia

**DOI:** 10.1186/s40793-015-0053-6

**Published:** 2015-10-08

**Authors:** Beng Soon Teh, Nyok-Sean Lau, Fui Ling Ng, Ahmad Yamin Abdul Rahman, Xuehua Wan, Jennifer A. Saito, Shaobin Hou, Aik-Hong Teh, Nazalan Najimudin, Maqsudul Alam

**Affiliations:** Centre for Chemical Biology, Universiti Sains Malaysia, Penang, Malaysia; School of Biological Sciences, Universiti Sains Malaysia, Penang, Malaysia; Advanced Studies in Genomics, Proteomics and Bioinformatics, University of Hawaii, Honolulu, Hawaii USA; Department of Microbiology, University of Hawaii, Honolulu, Hawaii USA; Present address: Department of Bioorganic Chemistry, Max Planck Institute for Chemical Ecology, Jena, Germany

**Keywords:** *Thermus*, Thermophile, Extremophile, Hot spring

## Abstract

*Thermus* sp. strain CCB_US3_UF1 is a thermophilic bacterium of the genus *Thermus*, a member of the family *Thermaceae*. Members of the genus *Thermus* have been widely used as a biological model for structural biology studies and to understand the mechanism of microbial adaptation under thermal environments. Here, we present the complete genome sequence of *Thermus* sp. CCB_US3_UF1 isolated from a hot spring in Malaysia, which is the fifth member of the genus *Thermus* with a completely sequenced and publicly available genome (Genbank date of release: December 2, 2011). *Thermus* sp. CCB_US3_UF1 has the third largest genome within the genus. The complete genome comprises of a chromosome of 2.26 Mb and a plasmid of 19.7 kb. The genome contains 2279 protein-coding and 54 RNA genes. In addition, its genome revealed potential pathways for the synthesis of secondary metabolites (isoprenoid) and pigments (carotenoid).

**Electronic supplementary material:**

The online version of this article (doi:10.1186/s40793-015-0053-6) contains supplementary material, which is available to authorized users.

## Introduction

*Thermus* spp. are Gram-negative, aerobic, non-sporulating, and rod-shaped thermophilic bacteria. *Thermus aquaticus* was the first bacterium of the genus *Thermus* that was discovered in several of the hot springs in Yellowstone National Park, United States [1]. A few years later, two strains of *Thermus thermophilus* (HB27 and HB8) were successfully isolated from thermal environments in Japan [2, 3]. To date, many strains of *Thermus* have been isolated from various geothermal environments such as hot springs and deep-sea hydrothermal vents. In addition to the ability to survive under thermal environments, *T. thermophilus* can also thrive in environments with extreme pH values, demonstrating great capabilities for adaptation to various environmental conditions.

The whole genome sequences of two strains of *T. thermophilus*, HB8 and HB27, were independently completed in 2004 [4, 5]. The genome of a second *Thermus* species, *Thermus scotoductus* SA-01, is also available [6]. *T. thermophilus* has attracted attention as one of the model organisms for structural biology studies because protein complexes from extremophiles are easier to crystallize than their mesophilic counterparts [7]. Some of the breakthrough examples of large complexes from thermophiles that have been crystallized are structures of the 70S ribosome [8], the bacterial RNA polymerase [9, 10] and the respiratory complex I [11] from *Thermus* spp. that were solved before those of *Escherichia coli*.

Members of the genus *Thermus* are of considerable biotechnological interest as sources of thermophilic enzymes [12, 13]. Thermozymes and proteins from the genus *Thermus* are good candidates for industrial processes because of their high thermal stability and co-solvent compatibility. The most well-known enzyme mined from the genus *Thermus* is DNA polymerase, an important enzyme used in PCR. Other than DNA polymerase, thermozymes from this genus are also widely used in food, pharmaceutical and paper-pulp industries [7]. Examples of industrial applications for thermostable enzymes include organic synthesis (*e.g.* esterases, lipases, proteases), starch-processing (*e.g.* α-amylases, glucose isomerases), pulp and paper manufacturing (*e.g.* xylanases) as well as animal feed and human food production (amino acid and vitamin synthesis) [13, 14]. Here, we present a summary of classification and a set of features for *Thermus* sp. CCB_US3_UF1, together with the description of the complete genome sequence and annotation.

## Organism information

### Classification and features

*Thermus* spp. are suggested to be closely related to the genus *Deinococcus* based on several comparative studies on 16S rRNA and protein sequences, and they form a distinct branch known as the *Deinococcus-Thermus* group [15, 16]. Nevertheless, the exact phylogenetic position of the *Deinococcus-Thermus* phylum remains to be determined. This phylum was proposed to derive from the oldest groups of the Bacteria Domain, after those of *Aquifex* and *Thermotoga* based on 16S rRNA sequence comparison [17]. A more in-depth analysis of the phylogeny of the *Deinococcus-Thermus* phylum based on conserved orthologs can be carried out as genome sequences from both of the genera are available [18].

**Fig. 1:**
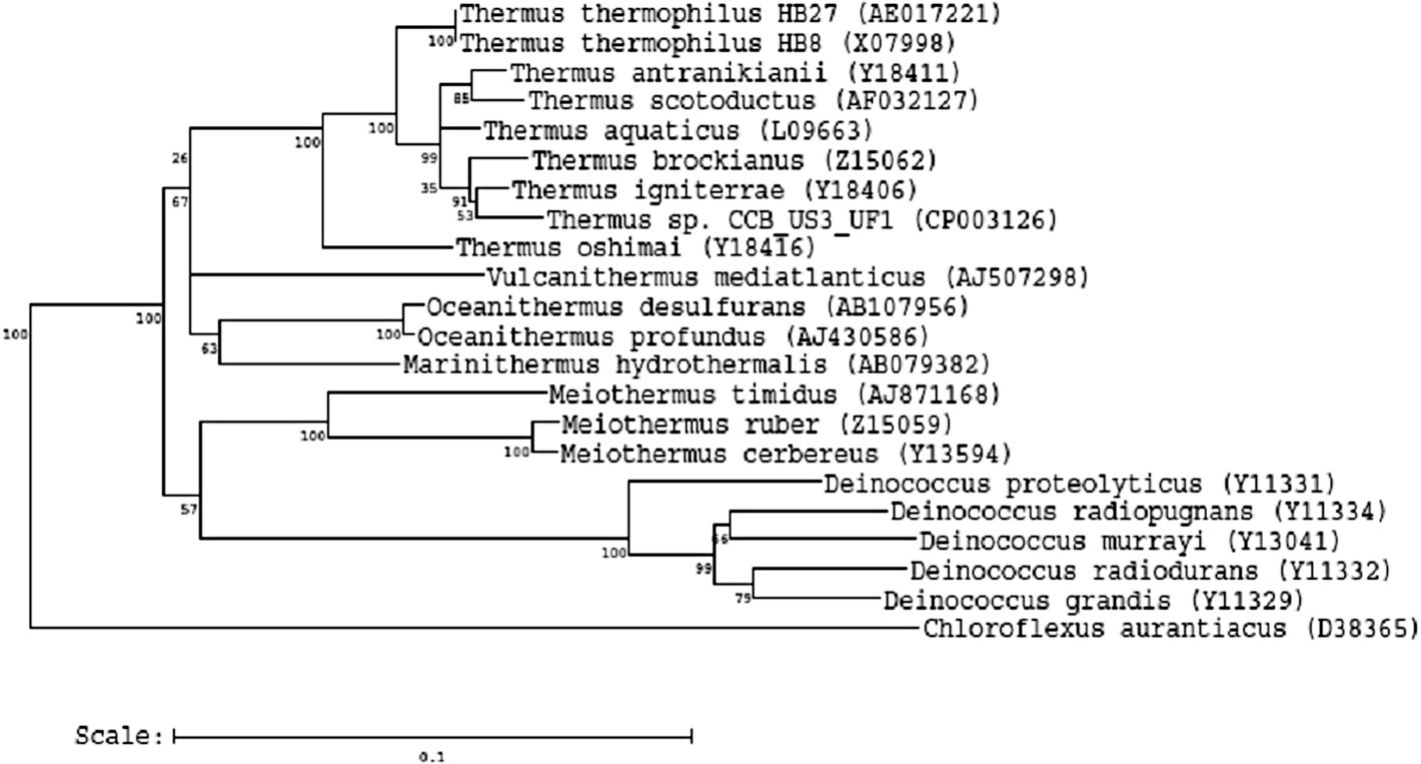
Phylogenetic tree highlighting the position of *Thermus* sp. CCB_US3_UF1 relative to the other type strains within the families *Deinococcaceae* and *Thermaceae*. Strains shown are those within the *Deinococcaceae* and *Thermaceae* having the corresponding NCBI genome project ids listed within [53]. The tree used sequences aligned by Ribosomal Database Project (RDP) aligner and Juke-Cantor corrected distance model. Distance matrix was constructed based on alignment model positions without the use of alignment insert, and a minimum comparable position of 200 was used. The tree was constructed with RDP Tree Builder that used Weighbor [54] with an alphabet size of 4 and a length size of 1000. The building of the tree involved a bootstrapping process that was repeated 100 times to generate a majority consensus tree [55]

In order to better understand the phylogeny of *Thermus* sp. CCB_US3_UF1, we constructed a phylogenetic tree based on the 16S rRNA gene sequences*.* There are two identical copies of the 16S rRNA gene in the *Thermus* sp. CCB_US3_UF1 genome. One copy of the gene sequence was used to search against the nucleotide database using NCBI BLASTN [19]. The BLASTN result shows that it has the highest sequence identity to *Thermus igniterrae* RF-4 (97 %, Y18406), *Thermus brockianus* YS38 (96 %, Z15062), and *Thermus scotoductus* Se-1 (95 %, AF032127). Figure 1 shows the phylogenetic neighborhood of *Thermus* sp. CCB_US3_UF1 relative to type strains of the families *Deinococcaceae* and *Thermaceae*. *Chloroflexus aurantiacus* (D38365) was used as an outgroup to root the tree.

**Fig. 2:**
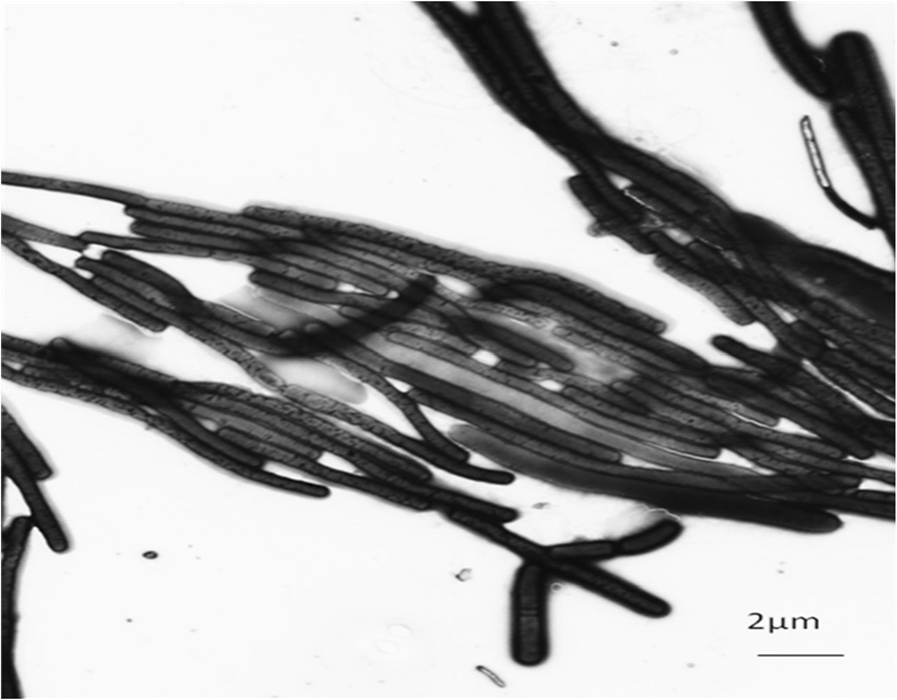
Transmission electron micrograph of *Thermus* sp. CCB_US3_UF1

*Thermus* sp. CCB_US3_UF1 is a Gram-negative bacterium (Table 1) and it has a rod-shaped filamentous structure (Fig. 2). Members of the genus *Thermus* are capable of growing at temperatures ranging between 45 °C and 83 °C [20]. Most of them have a maximum temperature for growth at slightly below 80 °C [21, 22]. Interestingly, a few strains of *T. thermophilus* can grow at 80 °C or above [23]. *Thermus* sp. CCB_US3_UF1 was isolated from a hot spring in Ulu Slim, Perak, Malaysia. It can grow well between 60 °C and 70 °C. *Thermus* spp. need carbohydrates, amino acids, carboxylic acids and peptides as sources of carbon and energy. The strain CCB_US3_UF1 is an aerobic, non-sporulating, non-motile and yellow-pigmented bacterium. Some of the members of the genus *Thermus* are capable of growing anaerobically using nitrate as an electron acceptor and some can even reduce nitrite [22, 23].

**Table 1:** Classification and general features of *Thermus* sp. CCB_US3_UF1 according to the MIGS recommendations [57]

MIGS ID	Property	Term	Evidence code^a^
	Classification	Domain *Bacteria*	TAS [17]
		Phylum *Deinococcus*-*Thermus*	TAS [58]
		Class *Deinococci*	TAS [59, 60]
		Order *Thermales*	TAS [60, 61]
		Family *Thermaceae*	TAS [60, 62]
		Genus *Thermus*	TAS [1, 63, 64]
		Species Unknown	IDA
		Type strain CCB_US3_UF1	IDA
	Gram stain	Negative	IDA
	Cell shape	Rod	IDA
	Motility	Non-motile	NAS
	Sporulation	Non-sporulating	NAS
	Temperature range	Thermophile (45-83 °C)	TAS [20]
	Optimum temperature	60 °C	IDA
	pH range; Optimum	Not reported	
	Carbon source	Not reported	
MIGS-6	Habitat	Hot springs	IDA
MIGS-6.3	Salinity	Not-reported	
MIGS-22	Oxygen requirement	Aerobic	NAS
MIGS-15	Biotic relationship	Free-living	NAS
MIGS-14	Pathogenicity	Non-pathogen	NAS
MIGS-4	Geographic location	Ulu Slim, Perak, Malaysia	IDA
MIGS-5	Sample collection	2009	IDA
MIGS-4.1	Latitude	3.898822°N	IDA
MIGS-4.2	Longitude	101.497911°E	IDA
MIGS-4.4	Altitude	51 m	IDA

^a^Evidence codes - *IDA* Inferred from Direct Assay, *TAS* Traceable Author Statement (i.e., a direct report exists in the literature), *NAS* Non-traceable Author Statement (i.e., not directly observed for the living, isolated sample, but based on a generally accepted property for the species, or anecdotal evidence). These evidence codes are from the Gene Ontology project [65]

## Genome sequencing and annotation

### Genome project history

The genus *Thermus* belongs to one of the oldest evolutionary branches of the Bacteria domain. The genome sequencing of *Thermus* sp. CCB_US3_UF1 was initiated as it can serve as a model bacterium for studying the evolution of thermophilic adaptation. The sequencing and finishing of the genome were completed at the Advanced Studies in Genomics, Proteomics and Bioinformatics (University of Hawaii) and TEDA School of Biological Sciences and Biotechnology (Nankai University, China). The genome annotation was performed at the Centre for Chemical Biology (Universiti Sains Malaysia). This genome sequence was first published in March 2012 [24]. A summary of the project information is shown in Table 2.

**Table 2:** Project information

MIGS ID	Project	Term
MIGS 31	Finishing quality	Finished
MIGS-28	Libraries used	Two genomic libraries: one 454 PE library (3 kb insert size), one Illumina library (3 kb insert size)
MIGS 29	Sequencing platforms	Illumina GA II×, 454 GS FLX Titanium
MIGS 31.2	Fold coverage	115× (Illumina); 21.14× (454)
MIGS 30	Assemblers	Newbler v 2.3, burrows-wheeler alignment (BWA)
MIGS 32	Gene calling method	Glimmer 3.02
	Locus tag	TCCBUS3UF1
	Genbank ID	CP003126, CP003127
	GenBank date of release	December 2, 2011
	GOLD ID	Gp0013444
	BIOPROJECT	PRJN76491
MIGS 13	Source material identifier	CCB_US3_UF1
	Project relevance	Biotechnology, pathway, extremophile

### Growth conditions and genomic DNA preparation

*Thermus* sp. CCB_US3_UF1 was grown aerobically to late exponential phase in 50 ml of ATCC medium 697 (*Thermus* medium) [3] at 60 °C. Genomic DNA was isolated from *Thermus* sp. CCB_US3_UF1 using a modified phenol-chloroform extraction protocol [25]. The quality of DNA was checked by 0.5 % agarose gel electrophoresis and its quantity by a NanoDrop 2000 Spectrophotometer (Thermo Scientific, Wilmington, Delaware, USA). A DNA concentration of 363.4 ng/μl and OD_260_/OD_280_ of 1.90 was obtained.

### Genome sequencing and assembly

The whole-genome sequencing of *Thermus* sp. CCB_US3_UF1 was performed using Roche 454 and Illumina paired-end sequencing technologies. A 3 kb genomic library was constructed and 97,991 paired-end reads and 54,397 single-end reads were generated using the GS FLX system, providing 21.14-fold genome coverage. Six large scaffolds including 51 contigs were successfully assembled from 97.09 % of the reads using the 454 Newbler assembly software (454 Life Sciences, Branford, CT). A total of 3,469,788 reads from 3 kb library were produced to reach a depth of 115-fold coverage with an Illumina GA IIx (Illumina, San Diego, CA). These reads were mapped to the scaffolds using the Burrows-Wheeler Alignment (BWA) tool [26]. The majority of the gaps within the scaffolds were filled by local assembly of 454 and Illumina reads. The gaps between the scaffolds were filled by sequencing PCR products using an ABI 3730xl capillary sequencer. PCR products were sequenced to verify repeats larger than 3 kb. The putative sequencing errors were verified and corrected by consensus of the Roche/454 and Illumina reads.

### Genome annotation

The automated annotation of the genome was done using the DIYA (Do-It-Yourself Annotator) pipeline [27]. The pipeline uses Glimmer3 to predict open reading frames [28], followed by protein similarity searches using BLAST [19] against UNIREF [29], RPS-BLAST against CDD [30], and Asgard [31]. In addition, RPS-BLAST searches against the COG database was done to enable assignment of COG functional categories to the ORFs. Transfer RNAs were predicted by using tRNAscan-SE [32] while ribosomal RNAs were identified by using RNAmmer [33].

## Genome properties

**Fig. 3:**
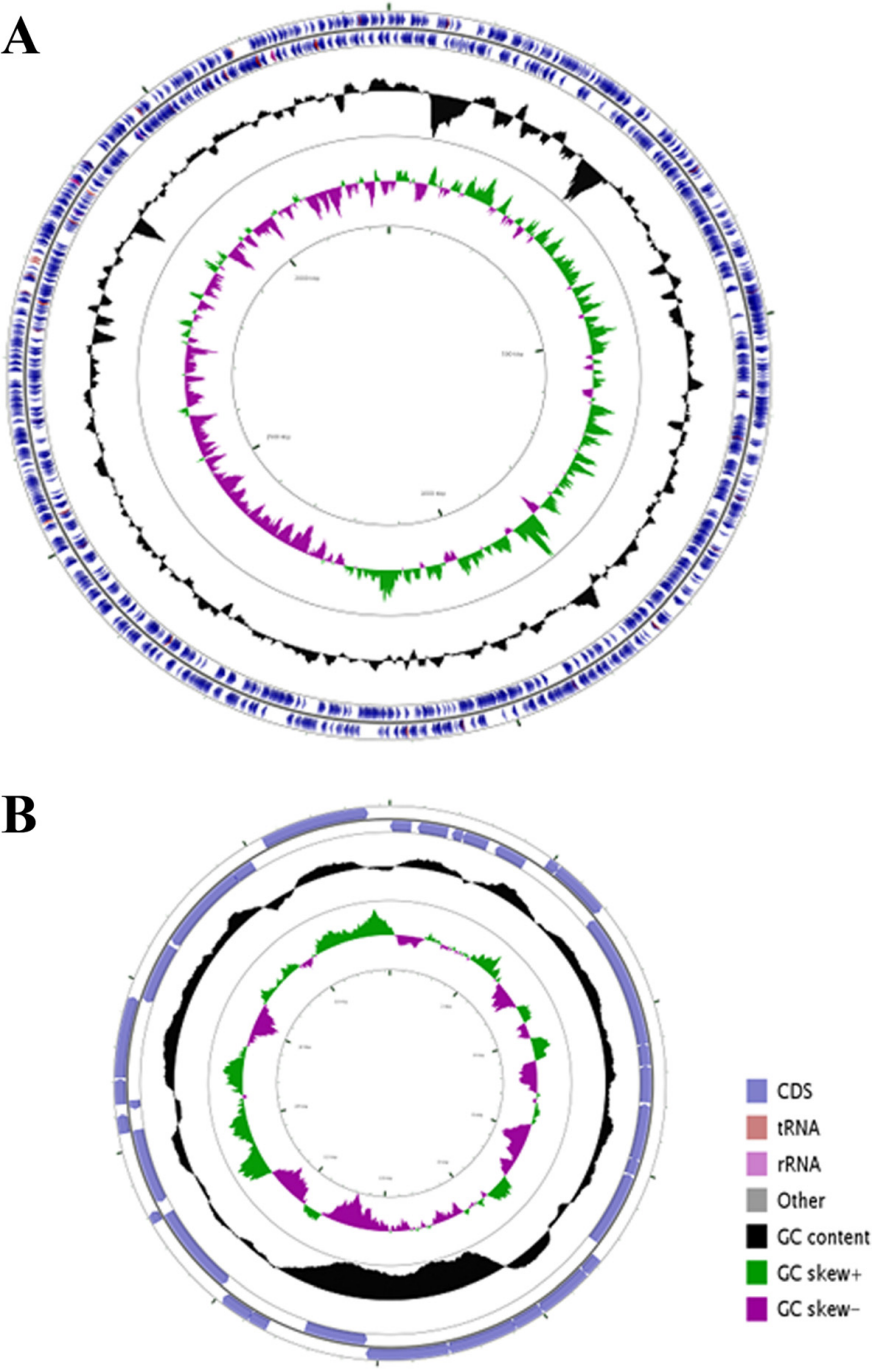
Graphical circular map of the *Thermus* sp. CCB_US3_UF1 chromosome and plasmid pTCCB09. **a** Chromosome. **b** Plasmid. From the inside to outside, the second and fourth circles show GC skew and G + C content respectively. The sixth and seventh circles show protein coding genes in positive and negative strands and RNA genes (tRNAs red, rRNAs light purple, other RNAs grey). This figure was generated by CGView [56]

The complete genome of *Thermus* sp. CCB_US3_UF1 is composed of a single circular chromosome of 2,243,772 bp and a plasmid of 19,716 bp with G + C contents of 68.6 % and 65.6 %, respectively (Fig. 3). There are 2334 predicted coding sequences (CDS), 2 rRNA operons, and 48 tRNA genes in the chromosome (Table 3). A total of 32 CDS are predicted in the plasmid. The distribution of genes into COG functional categories is presented in Table 4.

**Table 3:** Genome statistics

Attribute	Value	% of Total^a^
Genome size (bp)	2,263,488	100.00
DNA coding (bp)	2,137,656	94.44
DNA G + C (bp)	1,552,285	68.58
DNA scaffolds	1	100.00
Total genes^b^	2,333	100.00
Protein coding genes	2,279	97.64
RNA genes	54	2.31
Pseudo genes	1	0.04
Genes in internal clusters	822	36.07
Genes with function prediction	2,072	90.92
Genes assigned to COGs	2,098	89.89
Genes with Pfam domains	1,469	64.46
Genes with signal peptides	113	4.96
Genes with transmembrane helices	460	20.18
CRISPR repeats	8	0.34

^a^The total is based on either the size of the genome in base pairs or the total number of protein coding genes in the annotated genome

^b^Pseudogenes may also be counted as protein coding or RNA genes, so their number is not additive under the total gene count

**Table 4:** Number of genes associated with general COG functional categories

Code	Value	% age^a^	Description
J	147	6.4	Translation, ribosomal structure and biogenesis
A	23	1.0	RNA processing and modification
K	97	4.2	Transcription
L	115	5.0	Replication, recombination and repair
B	3	0.1	Chromatin structure and dynamics
D	38	1.6	Cell cycle control, cell division, chromosome partitioning
Y	0	0.0	Nuclear structure
V	27	1.2	Defense mechanisms
T	77	3.3	Signal transduction mechanisms
M	91	3.9	Cell wall/membrane biogenesis
N	63	2.7	Cell motility
Z	0	0.0	Cytoskeleton
W	0	0.0	Extracellular structures
U	50	2.2	Intracellular trafficking and secretion
O	90	3.9	Posttranslational modification, protein turnover, chaperones
C	145	6.3	Energy production and conversion
G	138	6.0	Carbohydrate transport and metabolism
E	247	10.7	Amino acid transport and metabolism
F	71	3.1	Nucleotide transport and metabolism
H	115	5.0	Coenzyme transport and metabolism
I	95	4.1	Lipid transport and metabolism
P	95	4.1	Inorganic ion transport and metabolism
Q	56	2.4	Secondary metabolites biosynthesis, transport and catabolism
R	310	13.4	General function prediction only
S	215	9.3	Function unknown
-	181	7.8	Not in COGs

^a^The total is based on the total number of protein coding genes in the genome

## Comparison with other sequenced genomes

The genome of *Thermus* sp. CCB_US3_UF1 (2.26 Mb) is larger than those of *T. thermophilus* HB27 (2.13 Mb) and *T. thermophilus* HB8 (2.12 Mb), but smaller than that of *T. scotoductus* SA-01 (2.36 Mb) (Table 5).

**Table 5:** Comparison of genome features of different species of *Thermus*

Species	*Thermus* sp. CCB_US3_UF1	*Thermus thermophilus* HB27	*Thermus thermophilus* HB8	*Thermus scotoductus* SA-01
Genome size (bp)	2,263,488	2,127,482	2,116,056	2,355,186
G + C content (%)	68.6	69.4	69.5	64.9
Number of protein coding genes	2,279	2,210	2,173	2,458
Coding area (%)	94.4	94.8	94.9	94.0
Total number of genes	2,333	2,263	2,226	2,511
Hypothetical genes	742	734	758	619
Proteins with assigned function	1,537	1,476	1,415	1,839
rRNA	6	6	6	6
tRNA	48	47	47	47
Transposase	13	18	18	22
CRISPR sequences	8	10	11	3

Table adapted from NCBI

The *Thermus* sp. CCB_US3_UF1 genome was compared against closely related *Thermus* genomes using BLAST and Artemis comparison tool to identify regions of synteny. The three closest *Thermus* with sequenced genomes (*T. thermophilus* strains HB27, HB8 and *T. scotoductus* SA-01) were selected for the comparison. The genome of strain HB27 consists of a chromosome (1.89 Mb) and a megaplasmid (0.23 Mb). On the other hand, strain HB8 has a chromosome of 1.85 Mb, a megaplasmid (0.26 Mb) and a plasmid (9.3 kb) [5]. The genome of *T. scotoductus* includes a 2.3 Mb chromosome and a plasmid of 8.4 kb.

*Thermus* sp. CCB_US3_UF1, *T. thermophilus* HB27, HB8 and *T. scotoductus* SA-01 all have a small genome size that is below 2.5 Mb. They also display a high G + C content that may correlate with their thermophilic lifestyle. CCB_US3_UF1 has a higher number of predicted protein coding sequences (2279) than HB27 (2210) and HB8 (2173), but lower than that of *T. scotoductus* SA-01 (2458). They also share a similar number of rRNA (16S-23S-5S) operons with a well-balanced high G + C content above 60 %, a common feature displayed by thermophilic bacteria. The number of tRNAs that are present in all four genomes is between 47 and 48. In terms of transposase genes, *T. scotoductus* SA-01 has the highest number (22 genes), followed by CCB_US3_UF1 (13 genes), *T. thermophilus* HB27 (18 genes), and HB8 (18 genes). Interestingly, no prophage-related genes are found in these four genomes, implying the occurrence of clustered regularly interspaced short palindromic repeats (CRISPRs). CRISPR is characterized as a type of antiviral immune system found in *Bacteria* and *Archaea* [34].

There are 1728 proteins, or 76 % of the total proteins, from *Thermus* sp. CCB_US3_UF1 that are found orthologous to the proteins in *T. thermophilus* HB27, and a total of 1691 (74 %) orthologs are shared between CCB_US3_UF1 and *T. thermophilus* HB8. Meanwhile, a total of 1885 (83 %) proteins are shared between CCB_US3_UF1 and *T. scotoductus* SA-01, showing greater similarity between these two species. The protein ortholog mapping was done with a cut-off e-value of 10^-5^ using the protein-protein BLAST (blastp). Despite of the similarity of many of their gene products, genome-wide synteny between *Thermus* sp. CCB_US3_UF1 and *T. thermophilus* HB27, HB8 and *T. scotoductus* SA-01 could not be detected.

The plasmid of *Thermus* sp. CCB_US3_UF1 shows no overall similarity to the other sequenced plasmids of *T. thermophilus* HB27 and HB8, but it has high similarity to the plasmid of *Thermus* sp. 4C, designated as pL4C [35]. The gene encoding chromosome segregation ATPase (TCCBUS3UF1_p160) that is found in pL4C is present in the *Thermus* sp. CCB_US3_UF1 plasmid. This protein has been suggested to play an essential role in plasmid replication and partition [36]. In addition, a putative integrase gene that facilitates gene transfer and chromosome modification can be found in both plasmids.

## Insights from the genome sequence

The *Thermus* sp. CCB_US3_UF1 genome encodes genes for complete tricarboxylic cycle, gluconeogenesis, glyoxylate bypass and Embden-Meyerhof pathways. Both *Thermus* sp. CCB_US3_UF1 and *T. thermophilus* HB27 share similar sets of genes that are involved in aerobic respiration. At high temperatures, the solubility of oxygen in water is low. Two terminal cytochrome oxidases are found in *Thermus*: a caa_3_- type (TCCBUS3UF1_540-550) that is expressed under high oxygen levels, and a ba_3_- type oxidase (TCCBUS3UF1_13990, TCCBUS3UF1_14010) that is expressed under low oxygen supply [37, 38]. *Thermus* sp. CCB_US3_UF1 is able to synthesize many important compounds, including amino acids, vitamins, cofactors, carriers, purines and pyrimidines. Many of these biosynthetic pathways show a high degree of conservation between CCB_US3_UF1 and *T. thermophilus* HB27. In addition, *Thermus* sp. CCB_US3_UF1 has branched-chain amino acid ABC transport systems that are important for nutrient acquisition, and ion transporters for the elimination of toxic compounds such as copper and arsenite.

### Motility and natural transformation

So far, motility is not observed in *Thermus* and no flagella biosynthetic gene is present in the genomes. However, genes encoding gliding motility proteins (TCCBUS3UF1_13970, TCCBUS3UF1_13980) and a twitching mobility protein (PilT, TCCBUS3UF1_9080) are found in the genome of *Thermus* sp. CCB_US3_UF1. These two proteins are also found in *T. thermophilus* HB27, HB8 and *T. scotoductus* SA-01, and this raises the question regarding the existence of motility in *Thermus*.

**Fig. 4:**
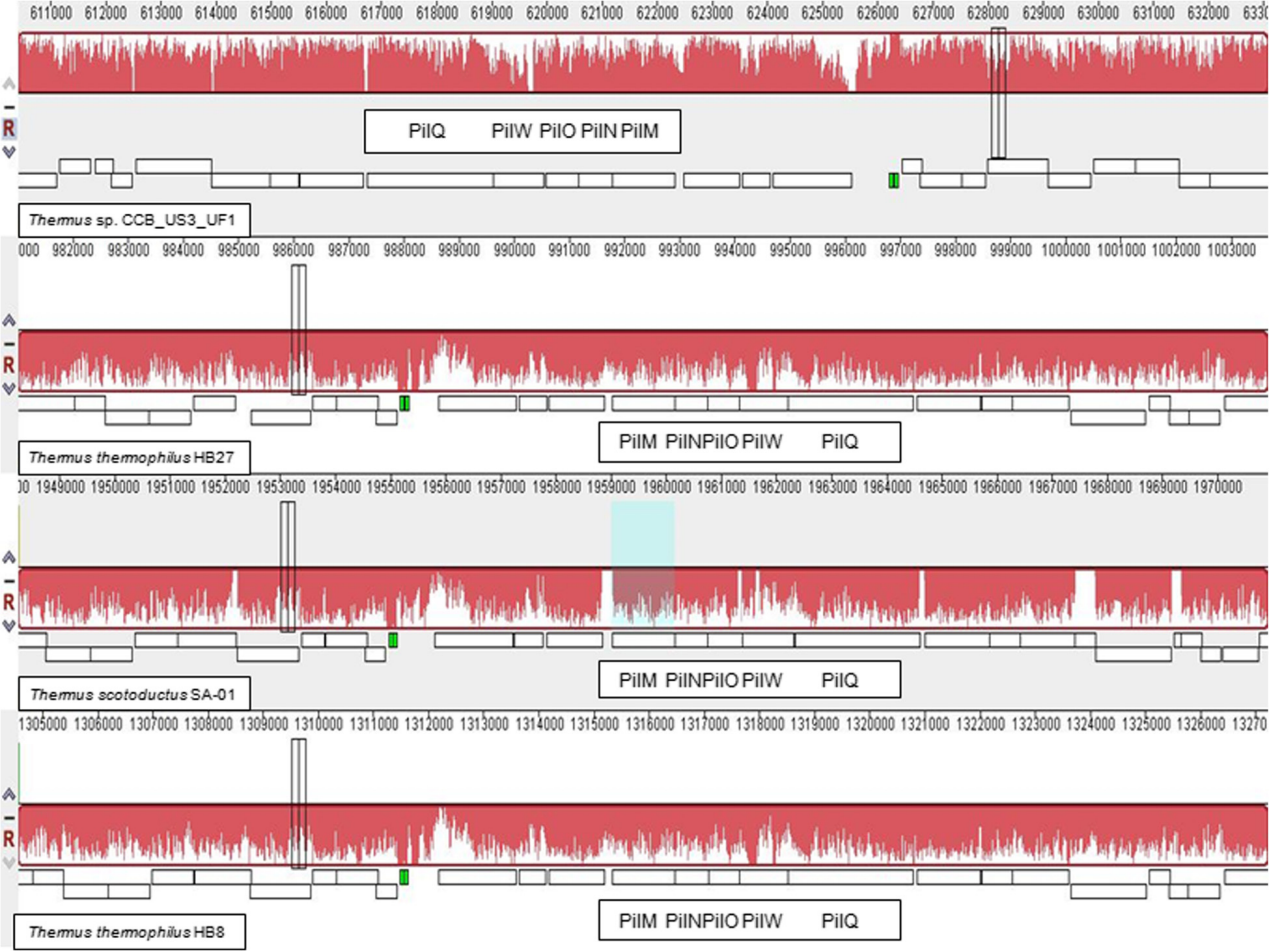
Comparison of competence proteins between *Thermus* sp. CCB_US3_UF1 and other *Thermus*-related species using MAUVE alignments

*Thermus* sp. CCB_US3_UF1 is also found to possess type IV pili that are crucial in the attachment, twitching motility, surface colonization, and natural transformation systems in bacteria [39]. The efficiency of a DNA uptake system in *T. thermophilus* is crucial to thermoadaptation and exchange of genetic materials in high temperature environments*.* Competence proteins play an important role in natural transformation and can be categorized into three groups: DNA-translocator-specific proteins, type IV pili (Tfp)-related proteins, and nonconserved proteins [40]. Genes encoding DNA-translocator-specific proteins [ComEA (TCCBUS3UF1_22560), ComEC (TCCBUS3UF1_22570), DprA (TCCBUS3UF1_18680)] and Tfp-related proteins [PilA1 (TCCBUS3UF1_8740), PilA2 (TCCBUS3UF1_8720), PilA3 (TCCBUS3UF1_8710)] were found in the *Thermus* sp. CCB_US3_UF1 genome. Genes encoding leader peptidase (PilD, TCCBUS3UF1_20930), traffic-NTPase (PilF, TCCBUS3UF1_21340), inner membrane protein (PilC, TCCBUS3UF1_8100), PilM-homolog and secretin-like protein (PilQ, TCCBUS3UF1_6320) were also identified. In addition, genes encoding competence proteins ComZ (TCCBUS3UF1_870), PilN (TCCBUS3UF1_6350), PilO (TCCBUS3UF1_6340), and PilW (TCCBUS3UF1_6330) were present in the chromosome of CCB_US3_UF1. The genes encoding PilM, PilN, PilO, PilW, and PilQ are found to cluster together in the genome (Fig. 4). The rearrangement of these genes is different in *Thermus* sp. CCB_US3_UF1 compared to *T. thermophilus* HB27, HB8 and *T. scotoductus* SA-01, demonstrating the loss of synteny between CCB_US3_UF1 and the other *Thermus* bacteria. The involvement of *Thermus* pili in DNA uptake has yet to be determined.

### Genomic islands

Potential genomic islands present in the *Thermus* sp. CCB_US3_UF1 genome were predicted using the IslandViewer database [41]. Early studies on genomic islands focused on regions that carry virulence factors and they are termed pathogenicity islands. Genomic islands are also shown to carry various types of genes associated with many metabolic pathways or biological processes [42]. A total of 11 possible genomic islands were identified in the *Thermus* sp. CCB_US3_UF1 genome. Several of these genomic islands carry genes encoding proteins involved in transport systems and defense mechanisms. For example, genomic islands 2, 3, 6 and 7 contain numerous transporter genes that may be involved in membrane transport in *Thermus* sp. CCB_US3_UF1. It is interesting to note that CRISPR-associated Cas proteins that are associated with phage immunity are present on Genomic Island 8 (245986 - 276477) and Genomic Island 10 (1323615 - 1334721). In comparison with other members of *Thermus*, *T. thermophilus* HB27 harbors 10 genomic islands while both *T. thermophilus* HB8 and *T. scotoductus* SA-01 carry 13 genomic islands.

### CRISPR

CRISPR is an RNAi-like system that provides adaptive immunity against phages or other infections is present in prokaryotes [43]. Using the CRISPR Finder tool [44], eight CRISPR repeat regions were detected in the *Thermus* sp. CCB_US3_UF1 genome (Table 6). The number of spacers in each of these loci are 3, 17, 14, 23, 18, 9, 12, and 2 respectively, i.e. a total of 98 spacers.

**Table 6:** Direct repeat consensus sequences of CRISPR loci

CRISPR locus	Direct repeat consensus
1	GTAGTCCCCACGCACGTGGGGATGGACC
2	GTTTCAAACCCTCATAGGTACGGTCAGAAC
3	CTTTGAACCGTACCTATAAGGGTTTGAAAC
4	CTTTGAACCGTACCTATAAGGGTTTGAAAC
5	GTTGCAAAAGTGGCTTCCCCGCAAGGGGATTGCGAC
6	GTCGCAATCCCCTTACGGGGAAGCCACTTTTGCAAC
7	GTCGCAATCCCCTTACGGGGAAGCCACTTTTGCAAC
8	CGTAGTCCCCACACGCGTGGGGATGGACC

A comparison with other *Thermus* sp. revealed that a total of 10, 11 and 3 CRISPRs were found in *T. thermophilus* HB27, HB8 and *T. scotoductus* SA-01, respectively. In terms of the number of spacers, *T. thermophilus* HB8 has the largest (112), followed by *Thermus* sp. CCB_US3_UF1 (98), *T. scotoductus* SA-01 (87) and *T. thermophilus* HB27 (74). The existence of a large number of CRISPRs in the *Thermus* genomes reflects an adaptation strategy employed by *Thermus* to protect themselves from foreign DNA invasion from the surrounding environments.

### Isoprenoid biosynthesis

Based on the genome information, *Thermus* sp. CCB_US3_UF1 synthesizes precursors for isoprenoid compounds from pyruvate and glyceraldehyde 3-phosphate using the deoxyxylulose phosphate (MEP/DOXP) pathway instead of the mevalonate pathway. Isoprenoid compounds are derived from the five-carbon precursor isopentenyl diphosphate (IPP). The genes encoding enzymes of the complete DOXP pathway could be identified in the genome. The DOXP pathway is initiated by the conversion of glyceraldehyde 3-phosphate and pyruvate to 1-deoxy-D-xylulose 5-phosphate (DOXP) catalyzed by DOXP synthase (TCCBUS3UF1_200). Isoprenoid synthesis then proceeds through a series of enzymatic reactions that lead to the formation of 2-C-methyl-D-erythritol-2,4-cyclodiphosphate. Genes encoding the enzymes involved are *dxr* (TCCBUS3UF1_15410), *ispD* (TCCBUS3UF1_19830), *ispE* (TCCBUS3UF1_19820), and *ispF* (TCCBUS3UF1_380). Genes *gcpE* (TCCBUS3UF1_18880) and *lytB* (TCCBUS3UF1_22000), which encode the enzymes involved in the last two steps of isoprenoid synthesis that lead to IPP formation are also encoded in the genome (Additional file 1: Figure S1).

### Carotenoid biosynthesis

Most species of the genus *Thermus* are characterized by the ability to synthesize yellow carotenoid-like pigments [45]. Carotenoids are natural pigments that have been used commercially as food colorants, nutrient supplements and for pharmaceuticals purposes [46]. *T. thermophilus* has been shown to produce carotenoids known as thermozeaxanthins and thermobiszeaxanthins [47]. As carotenoids are one of the hydrophobic components associated with the cell membrane, it was suggested that carotenoids might have an essential role in stabilizing the membrane of *Thermus* at high temperature. In *T. thermophilus* HB27, genes encoding the terminal steps of carotenoid biosynthesis are found in the large plasmid (pTT27), whereas precursor synthesis involving the formation of geranylgeranyl pyrophosphate (GGPP) is accomplished by enzymes encoded on the chromosome [5]. In *Thermus* sp. CCB_US3_UF1, genes encoding the enzymes for both the terminal and precursor steps of carotenoid biosynthesis are located on the chromosome (Additional file 2: Figure S2).

In the bacterial carotenoid biosynthetic pathway, phytoene is the first carotenoid synthesized and it is formed from the condensation of two molecules of geranylgeranyl pyrophosphate (GGPP) [48]. The GGPP synthase gene (TTHA0013) from *T. thermophilus* HB8 has been identified and functionally characterized [49]. The gene has a homolog (TCCBUS3UF1_18840) in *Thermus* sp. CCB_US3_UF1. Phytoene is synthesized from GGPP by phytoene synthase (CrtB). In *T. thermophilus* HB27, a gene encoding a homolog of phytoene synthase (TT_P0057) was cloned and identified as *crtB.* It was suggested that phytoene synthase is the rate-limiting enzyme in the carotenoid biosynthesis in *T. thermophilus*. In addition, *crtB* of *T. thermophilus* was found to cluster together with other carotenogenic genes on the large plasmid [50]. Interestingly, the homolog of *crtB* (TCCBUS3UF1_10160) in *Thermus* sp. CCB_US3_UF1 is encoded on the chromosome and not the plasmid. Phytoene is then converted to lycopene via a series of desaturation steps that are catalyzed by phytoene desaturase (CrtI), cis-carotene isomerase (CrtH) and ζ-carotene desaturase [51]. In *Deinococcus-Thermus* bacteria, only one phytoene desaturase, CrtI, has been detected. A gene encoding a CrtI homolog (TCCBUS3UF1_10090) is detected in the genome of *Thermus* sp. CCB_US3_UF1 as well [5, 52]. Following lycopene synthesis, the carotenoid biosynthetic pathway branches into acyclic and cyclic carotenoids formation. A possible gene encoding an enzyme that catalyzes the cyclization of lycopene, CrtY-type lycopene cyclase (TCCBUS3UF1_10120) is found in the genome of *Thermus* sp. CCB_US3_UF1 and the other three sequenced *Thermus* genomes [52] (Additional file 2: Figure S2).

## Conclusion

*Thermus* have proven to be useful as sources of thermostable enzymes and the genome sequences provide information for further exploring the biotechnological potentials of this genus. Analysis of the *Thermus* sp. CCB_US3_UF1 genome revealed that it encodes pathways for the synthesis of secondary metabolites (isoprenoid) and pigments (carotenoid). The latter has attracted industrial interest for application in food industries. The CRISPR/ Cas system that is found in *Thermus* could be an interesting tool in molecular biology, particularly for genome editing. Considering the great potential of *Thermus* in various fields, the complete genome sequence of *Thermus* sp. CCB_US3_UF1 is a valuable resource for both fundamental researches and biotechnological applications.

## Additional files

Additional file 1: Figure S1. Metabolic pathway reconstruction of isoprenoid biosynthesis. (TIFF 2139 kb) Additional file 2: Figure S2. Metabolic pathway reconstruction of carotenoid biosynthesis. (TIFF 2137 kb)

## Abbreviations

CCB: Centre for chemical biology
TEDA: Tianjin economic-technological developmental area
ATCC: American type culture collection
CRISPR: Clustered regularly interspaced short palindromic repeats
BWA: Burrows-wheeler alignment
USM: Universiti Sains Malaysia
