# Supplementary figures and images for: Complete genome sequence of the thermophilic Thermus sp. CCB_US3_UF1 from a hot spring in Malaysia
Source: Stand Genomic Sci. 2015 Oct 8;10:76. doi: 10.1186/s40793-015-0053-6 (PMC4599208; doi:10.1186/s40793-015-0053-6)

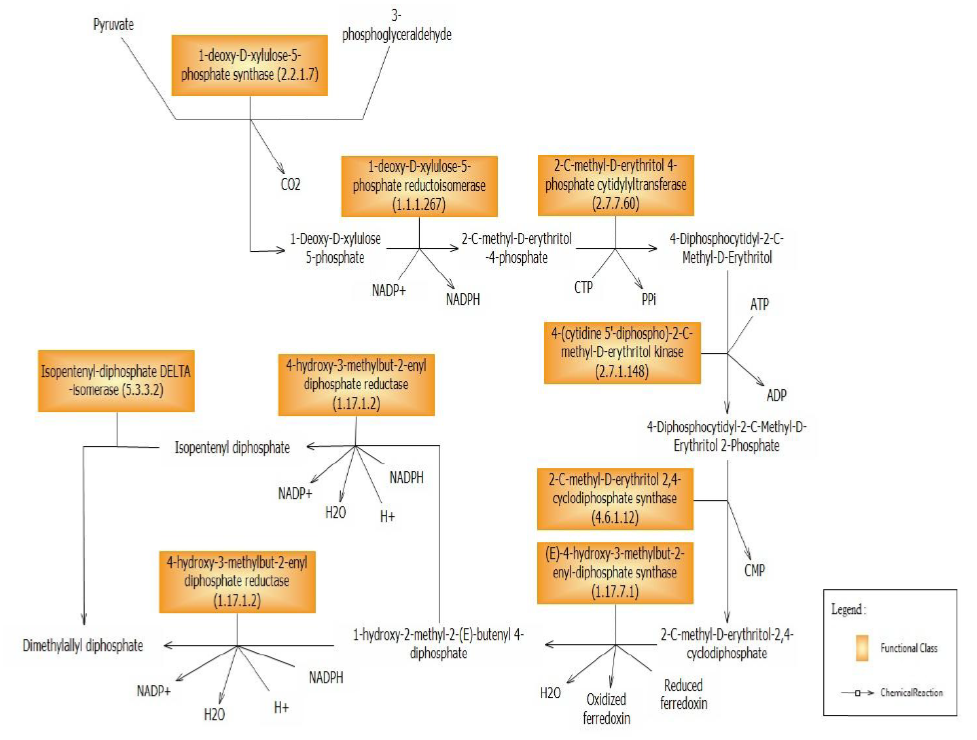

Supplement: Additional file 1: Figure S1. — Metabolic pathway reconstruction of isoprenoid biosynthesis. (TIFF 2139 kb) [file 40793_2015_53_MOESM1_ESM.tiff]

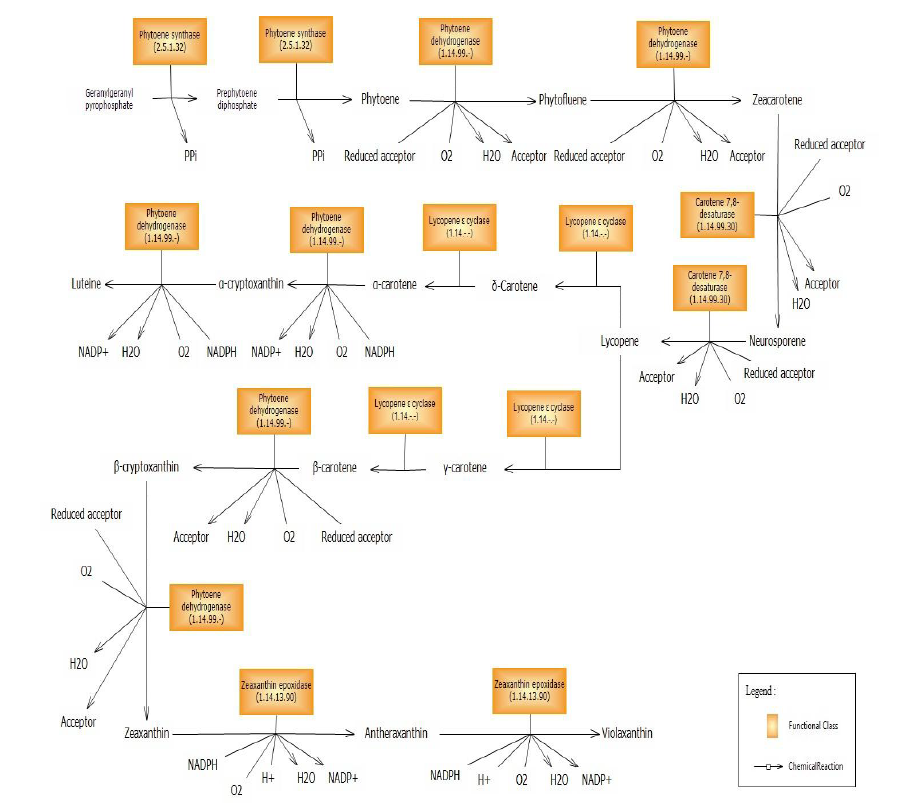

Supplement: Additional file 2: Figure S2. — Metabolic pathway reconstruction of carotenoid biosynthesis. (TIFF 2137 kb) [file 40793_2015_53_MOESM2_ESM.tiff]
